# Systemic Regulation of Cancer Development by Neuro-Endocrine-Immune Signaling Network at Multiple Levels

**DOI:** 10.3389/fcell.2020.586757

**Published:** 2020-09-30

**Authors:** Shu-Heng Jiang, Xiao-Xin Zhang, Li-Peng Hu, Xu Wang, Qing Li, Xue-Li Zhang, Jun Li, Jian-Ren Gu, Zhi-Gang Zhang

**Affiliations:** State Key Laboratory of Oncogenes and Related Genes, Shanghai Cancer Institute, Ren Ji Hospital, School of Medicine, Shanghai Jiao Tong University, Shanghai, China

**Keywords:** systematic regulation, neurotransmitter, inter-organ communication, immune evasion, chronic stress, perineural invasion

## Abstract

The overarching view of current tumor therapies simplifies cancer to a cell-biology problem in which neoplasms are caused solely by malignant cells and the exploration of carcinogenesis and tumor progression largely focuses on somatic mutations and other genetic abnormalities of cancer cells. The limited therapeutic response indicates that cancer is driven not only by endogenous oncogenic factors and reciprocal interactions within the tumor microenvironment, but also by complex systemic processes. Homeostasis is the fundamental premise of health, and is maintained by systemic regulation of neuro-endocrine-immune axis. Cancer is also a systemic disease that manifested by dysfunction of the nervous, endocrine, and immune systems. Multiple axes of regulation exist in cancer, including central-, organ-, and microenvironment-level manipulation. At each specific regulatory level, the tridirectional communication among the nervous, endocrine, and immune factors transmit flexible signaling to induce proliferation, invasion, reprogrammed metabolism, therapeutic resistance, and other malignant phenotypes of cancer cells, resulting in the extremely poor prognosis of this lethal disease. Understanding this coordinated signaling network will enable the development of new approaches for cancer treatment via behavioral and pharmacological interventions.

## Introduction

In the past several decades, therapeutic options for cancer have been developed from surgery, chemotherapy and radiotherapy to targeted therapy and immunotherapy. However, the prognosis of cancers remains tremendously unsatisfying and the death rate is still high (Siegel et al., 2017). Herein, the vast investments in cancer research and unsatisfactory status of treatment have highlighted a question: what is the nature of cancer? The traditional view of solid tumors is the autonomous abnormal growth of heterogeneous populations of neoplastic cells in local tissues. The tumor microenvironment (TME) has emerged as an equally important determinant of malignant tumor behaviors. However, there is still no space to explain how psychosocial and neuroendocrine factors might affect tumor initiation and development.

Host homeostasis is maintained by perfect interactions between the nervous, endocrine and immune systems involving different glands and organs. The neuro-endocrine-immune (NEI) network, first proposed by Besedovsky and Sorkin (1977), acts as an integrated unit to optimize health and develop defense against various complex pathological processes. Reciprocal influences have been eloquently evidenced among the NEI network; neurotransmitters, neuropeptides, and hormones affect immune functions, and immune cell products can affect neuro-endocrine mechanisms. Alteration of any system components or loss of integrated connections appears to be a marker of disease risk, especially for cancers. Psychological factors such as stress, anxiety and depression, endocrine factors and immunological mediators are closely associated with tumor initiation and development. Therefore, the NEI network may be a potentially ideal index reflecting cancer diseases.

In this review, we propose that cancer is a complicated, flexible and systemic disease encompassing multiple dysregulated process within the NEI system, including dysregulation at the central-, organ-, and TME levels. Then, emerging evidence regarding the role of the NEI system at each regulatory level is summarized. Last, given the role of the NEI signaling network in dictating the malignant phenotype, opportunities for developing innovative therapeutic approaches are discussed.

## Cancer Is a Systemic Disease

### Nervous System and Cancer

**FIGURE 1:**
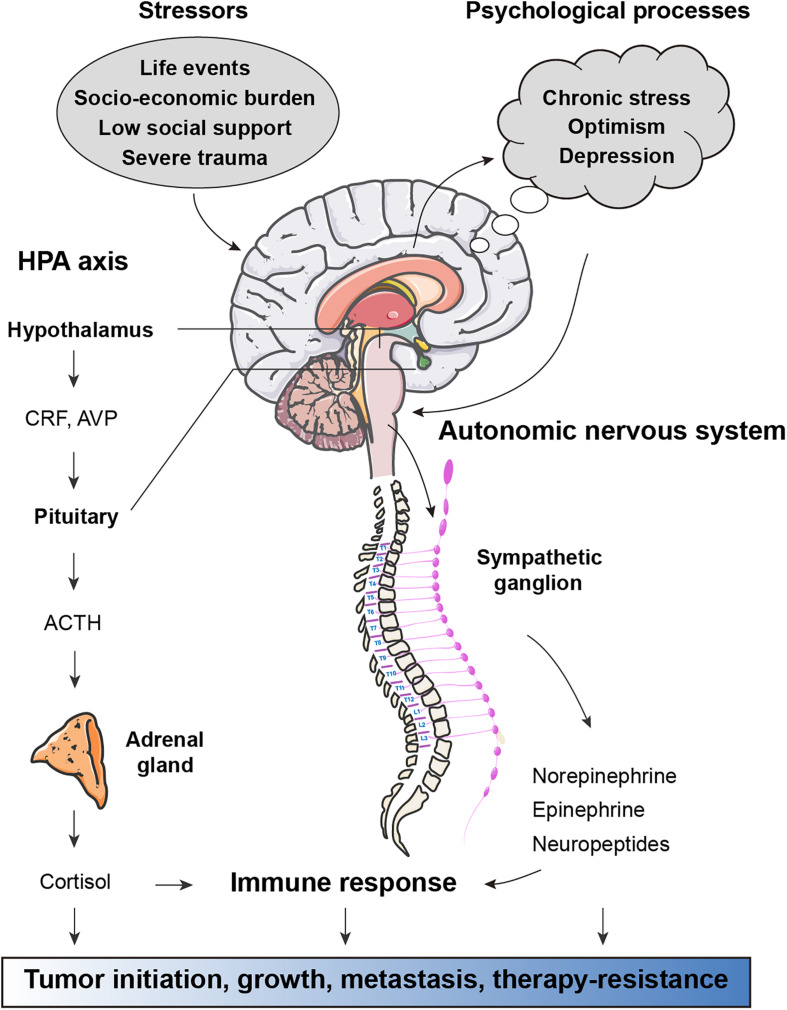
Effects of stress-associated factors on cancer pathogenesis. Stressful events result in activation of cortical and limbic structures of the central nervous system, which in turn activate the hypothalamic–pituitary–adrenal (HPA) axis and autonomic nervous system (ANS). In response to psychological stress, the paraventricular nucleus of the hypothalamus secretes corticotrophin-releasing hormone (CRH) and arginine vasopressin (AVP), both of which stimulate the anterior pituitary to release adrenocorticotrophic hormone (ACTH). Circulating ACTH activates the adrenal cortex to produce glucocorticoid hormone cortisol. ANS responses to stress are largely mediated by activation of the sympathetic nervous system (SNS) subsequent secretion of epinephrine (E) and norepinephrine (NE). Factors released from HPA and ANS axis have direct effects on cancer pathogenesis. Moreover, glucocorticoids, catecholamines, and other neuroendocrine factors can influence immune response to support tumor initiation, growth, metastasis, and therapy-resistance.

The question of whether physical dysfunction and mental disorders are associated with the prevalence, incidence and outcome of cancers has attracted much attention for centuries. External psychosocial processes activate cortical and limbic structures of the central nervous system, which subsequently ultimately activate the hypothalamic–pituitary–adrenal (HPA) axis and sympathetic nervous system (SNS) to trigger defeat/withdrawal responses and fight-or-flight stress responses, respectively (Antoni et al., 2006) (Figure 1). In the HPA axis, corticotrophin-releasing hormone is secreted from the paraventricular nucleus of the hypothalamus, stimulating the anterior pituitary to produce adrenocorticotrophic hormone, which in turn induces the downstream release of the glucocorticoid hormone cortisol from the adrenal cortex. Similarly, activation of the SNS induces the secretion of norepinephrine (NE) and epinephrine (E) from the adrenal medulla. There is strong evidence for links between alterations in neuroendocrine dynamics and tumor pathogenesis (Lutgendorf et al., 2010). Neuroendocrine factors, particularly catecholamines and cortisol, have been shown to have modulatory effects on immune processes related to tumor surveillance (Reiche et al., 2004). In preclinical models, chronic stress is sufficient to promote tumor growth and angiogenesis in ovarian carcinoma (Thaker et al., 2006), breast cancer lung colonization (Chen et al., 2018), EGFR inhibitor resistance in non-small cell lung cancer (NSCLC) (Nilsson et al., 2017), and therapy-induced anticancer immunosurveillance (Yang H. et al., 2019). In contrast, positive factors such as physical activity and optimism are known to reduce the risks of many human cancer types and predict longer survival (Allison et al., 2003; Moore et al., 2016). Thus, mental health, a key component of systemic regulation of physical function, is a key element for cancer initiation and progression.

### Endocrine and Cancer

Emerging persuasive evidence has revealed that systemic diseases, such as endocrine disorders and metabolic alterations, are involved in the development of non-endocrine tumors. Disrupted endocrine rhythms induced by stress or circadian deregulation are known to favor tumorigenesis (Antoni et al., 2006). Apart from the known stress-related hormones (glucocorticoids, prolactin, oxytocin, and dopamine), abnormalities in thyrotropin-releasing hormone (TRH), thyroid-stimulating hormone (TSH), and growth hormone (GH) have also been found in several cancer types such as colon cancer, gastric cancer, and lung cancer (Kamijo et al., 1987; Mazzoccoli et al., 2010). Moreover, epidemiological data and mechanistic studies have well documented the causal association between obesity and cancers (Renehan et al., 2015; Iyengar et al., 2016). A comparative risk assessment showed that diabetes and high BMI (BMI greater than or equal to 25 kg/m^2^) related to approximately 4.5% of all incident cancers. Diabetes and high BMI are associated with 25.8% and 31.9% increases in cancer prevalence respectively as risk factors (Pearson-Stuttard et al., 2018). The global burden from cancers, including liver, breast, endometrial, gallbladder and pancreatic cancer, are attributable to diabetes (Tsilidis et al., 2015). Intriguingly, prevailing concepts have recognized obesity as a spectrum of neuropsychological diseases that, are influenced by stress-related psychosocial factors and addiction-like behavior, rather than just an endocrine disease, suggesting close neuro-endocrine interactions (Jauch-Chara and Oltmanns, 2014; Niccolai et al., 2019).

### Immunity and Cancer

The importance of the immune system in cancer development has been greatly appreciated and immunotherapy is one of the most promising strategies for cancer treatment, which has been elegantly reviewed elsewhere (Galluzzi et al., 2018; June et al., 2018). Immune cells receive and respond to signals from neurotransmitters and hormones. Neuroendocrine mediators such as adenosine are profoundly implicated in immunosuppressive mechanisms in cancers (Vijayan et al., 2017). Essentially, neuroendocrine and immune factors fuel one another. Stress exposure causes a purine metabolic disorder in CD4^+^ T cells and induces anxiety-like behavior (Fan et al., 2019); innate immune cells affect neurotransmitter metabolism, neuronal endocrine function, and neuroplasticity by secreting cytokines (Engler et al., 2017; McKim et al., 2018). Moreover, depression can prime larger cytokine responses to pathogens or stressors; chronic stress, obesity, and sleep disorders contribute to prolonged and unrestrained inflammatory responses, which further facilitate the development of cancer (Del Rey and Besedovsky, 2017).

### Neuro-Endocrine-Immune-Related Genes and Cancer

Molecular evidence supports that the aberrantly expressed genes involved in systemic regulation also contribute to malignancy. To elucidate the function of genes in cancer development and progression, we previously performed a large-scale cDNA transfection screen and found 2,836 genes involved in cell growth and survival (Wan et al., 2004). These genes are categorized into four groups: I, genes related to basic cellular activities for cell survival and growth; II, genes participating in components of the TME; III, genes involved in host-cell systemic regulation and homeostasis; and IV, genes with unknown functions in cell survival. Among them, the third category of genes, which are related to host-cell systemic regulation, attracted our attention. These genes regulate four types of physiological activities: I, response to environmental changes (nutrition and redox); II, immune reaction (for example, cytokine/chemokine receptors); III, cellular electrophysiology (ion channels, exchange transporters, and small molecule transporters); and IV, nervous and hormonal regulation (neurotransmitter receptors, hormone receptors, insulin receptors, and progesterone receptors).

Collectively, diverse physical or pathological manifestations can result from central nervous system regulation, depending on the interconnected associations among nervous system, hormonal, and immune regulators. Hence, we presume that cancer is a systemic disease characterized by uncontrolled proliferation in local lesions (liver, stomach, pancreas, breast, prostate, etc.). This concept describes a systemic regulation of both “microscopic” and “macroscopic” aspects, including dysregulation of the NEI network at central, organ, and TME levels.

## Regulation at the Central Level

### Negative Stress Response and Cancer

The association between psychological and physiological factors of cancer risk and progression has been prevailingly confirmed in the past several decades. Chronic stress, caused by the persistent activation of the HPA system and the SNS, is involved in the development, progression, and mortality of various types of malignant cancer (Russell and Lightman, 2019). A meta-analysis comprehensively evaluating 165 studies referring to multiple kinds of cancer indicated that stress-related psychosocial factors are closely related to higher cancer incidence; meanwhile, poor prognosis and survival of diagnosed patients are showed in 330 studies, and high mortality is noted in 53 studies (Chida et al., 2008). A study of 281,290 individual participants showed that work stress is significantly associated with the risk of colorectal, lung, and esophageal cancers (Yang T. et al., 2019). Moreover, stressful life events, such as threats, death of close family members, and financial difficulties, have been proposed to contribute to the etiology of breast cancer (Lillberg et al., 2003). Studies in preclinical models have provided compelling evidence regarding the effects of chronic stress on tumorigenesis and the underlying molecular mechanisms. For instance, chronic neuropsychological stress promotes Kras-induced pancreatic tumorigenesis, driven by activated β2-adrenergic receptor signaling, nerve growth factor secretion, and upregulation of catecholamines. A successful combination therapy using tropomyosin receptor kinase inhibitor with gemcitabine was described in a pancreatic cancer animal model, but the rationale and mechanistic insight on the successful combination was not provided (Renz et al., 2018a). In ovarian carcinoma, a pioneering study showed that behavioral stress enhances tumor angiogenesis *in vivo* and thereby promotes malignant cell growth, which resulted from aberrant β-adrenergic activation of the cAMP-PKA signaling pathway (Thaker et al., 2006). In addition to tumorigenesis, mental stress has been proven to aggravate drug resistance. The cooperative signal between stress hormones activating β2-adrenergic receptors and mutant EGFR results in inactivation of liver kinase B1 (LKB1), a tumor suppressor, and upregulation of IL-6, which leads to tyrosine kinase inhibitor (TKI) resistance in a T790M-independent manner in the treatment of NSCLC (Nilsson et al., 2017), suggesting that combinations of the well characterized and safely administered β-blockers with EGFR TKIs merit further investigation as a strategy to overcome drug resistance. Chronic stress is known to decrease cellular immunity and immunosurveillance. Recent experimental studies have elucidated that stress-induced glucocorticoid surge and *Tsc22d3* upregulation in dendritic cells subvert chemotherapy or immunotherapy-induced anticancer immunosurveillance; in cancer patients, there is a close correlation among plasma cortisol levels, TSC22D3 expression in circulating leukocytes and negative mood (Yang H. et al., 2019). However, more works are warranted to determine the effects of chronic stress on other immune cells and to constitute actionable therapeutic targets.

### Positive Stress Response and Cancer

On the other hand, eustress stimulation and positive factors are efficient to hinder tumor growth. Environmental enrichment (EE) is a component of animal husbandry that aims to enhance sensory, cognitive, motor, and social stimuli, which are considered necessary for the optimal psychological and physiological well-being of animals. Mice living in the enriched housing environment showed less tumor growth and more frequent remission in melanoma and colorectal cancer, caused by elevated serum brain-derived neurotrophic factor (BDNF) and markedly lower leptin concentrations (Cao et al., 2010). Interestingly, EE inhibits mammary tumor growth rate with intact leptin signaling in diet-induced obesity models (Foglesong et al., 2019). In pancreatic cancer, EE was reported to significantly reduce tumor weight in subcutaneous and orthotopic mouse models via down-regulation of the expression of mitochondria-related genes (Li et al., 2015). Mechanistically, the modulation and enhancement of immunosurveillance and immune defense by EE is a robust player in anticancer events. EE exerts its protective effects through elevating the ratio of CD8^+^ cytotoxic T lymphocytes (CTL); because both propranolol and mifepristone can block the EE-associated modulation of CTLs, suggesting that both SNS and HPA axis are involved (Xiao et al., 2016). In most conditions, natural killer (NK) cells play a predominant role in the tumor inhibition induced by EE exposure (Garofalo et al., 2015; Song et al., 2017; Takai et al., 2019). Consistently, observations from glioma studies revealed that depletion of NK cells reduces the anti-tumor efficacy of EE (Garofalo et al., 2015). Additionally, EE exposure also induces microglia/macrophage (M/Mφ) activation, suggesting the diverse immunomodulatory effects of EE in cancers. Excitingly, EE was recently found to have an additive effect with the immune checkpoint therapy against tumor development (Watanabe et al., 2020).

In humans, a higher degree of optimism is associated with a lower cancer-related mortality. For instance, optimistic individuals have a 16% lower hazard ratio for all cancers compared with pessimistic individuals (Kim et al., 2017). In parallel, leisure-time physical activity is associated with conceivable anti-tumor effects through sympathetic activation, reduced endocrine factors (sex hormones, insulin, and myokines), and improved immune function (Hojman et al., 2018). Therefore, NEI regulation couples exercise to cancer prevention and treatment. In summary, at the central-regulation level, the interruption of neuro-hormone-immune balance is a vital driver of malignant tumors. It is still an open question whether EE has additional physiological functions apart from its effects on the neuro-hormone-immune networks.

## Regulation at the Organ Level

### Inter-Organ Signaling Communication

Many physiological functions and pathological initiation and progression originate from communication between organs, which occurs via hormones and cytokines, produced by both peripheral organs and the central nervous system (Karsenty and Olson, 2016; Castillo-Armengol et al., 2019). Two excellent examples are inter-organ growth coordination: growth-impaired organs induce the systemic growth inhibition of undamaged organs by producing Dilp8 hormone (Boulan et al., 2019), and endocrine-regulated metabolic homeostasis (Scopelliti et al., 2019). Moreover, the crosstalk between the liver and intestine by FGF19 performs important functions in cholesterol metabolism and energy homeostasis (Castillo-Armengol et al., 2019). Lipid metabolites work as long-range hormones to transmit signals around several organs, including liver, muscle, and adipose tissue, to regulate systemic energy metabolism and immune metabolism (Liu et al., 2014). Intriguingly, the endocrine capability is not restrained to traditional endocrine organs (such as the pancreas, liver, and adrenal gland). Bone has been uncovered to affect the pancreas, testes, and brain by secreting osteocalcin; natriuretic peptides released from the heart stimulate fatty acid oxidation in liver and white adipose tissue (Karsenty and Olson, 2016). Dysregulation of inter-organ interactions can exist as a reason or predictor for certain diseases. For example, hepatic steatosis has a casual role in the progression of insulin resistance in other tissues, such as skeletal muscles; steatotic hepatocytes release a kind of secreted protein, hepatokine, which puts dramatically impairment to liver (by promoting insulin resistance and injuring glucose effectiveness) and distant organs (including skeletal muscles, pancreas, adipose tissue, immune cells, and blood vessels) (Meex and Watt, 2017). Therefore, deciphering the molecular mechanism of these inter-organ communications is helpful for redefining therapeutic strategies to fight metabolic disorders and other related diseases.

### Inter-Organ Signaling and Cancer

**FIGURE 2:**
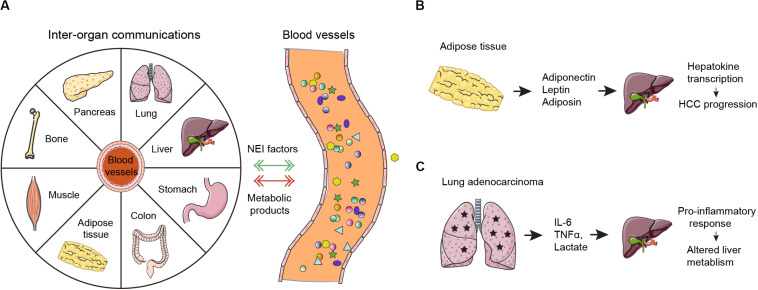
Neuro-endocrine-immune factors mediates tumor-organ communication. **(A)** In normal conditions, inter-organ communications are mediated primarily by neuro-endocrine-immune (NEI) factors. In cancers, the tumor microenvironment-derived endocrine hormones, immune factors (cytokines and chemokines) and metabolic byproducts (such as lactate) are secreted into the blood. These neuro-endocrine-immune factors can connect systemic metabolism and induce metabolic alterations in many human organs including the liver, the pancreas, adipose tissue, skeletal muscle and others as shown. Moreover, peripheral organs further facilitate tumorigenesis by potentially supplying metabolic fuel. **(B)** Adiponectin, leptin, and adiposin are secreted by adipose tissues. Then, they target the liver to increase hepatokine transcription, which ultimately promotes HCC progression. **(C)** Lung adenocarcinoma derived IL-6, TNFα, and lactate target the liver to induce pro-inflammatory response and alter the liver metabolism.

Inter-organ communication is also profoundly implicated in tumorigenesis (Figure 2A). For example, cytokines and extracellular vesicles secreted from adipose tissue can be transported to liver tissues and induce changes in hepatokine transcription and autophagy processes, which ultimately contribute to hepatocellular carcinoma development (Figure 2B). Growing evidence suggests that obesity has been assigned with specific roles in neural regulation, endocrine regulation, and immune responses in tumor progression. First, obesity-induced changes prompt imbalances in sympathetic-parasympathetic activity, which might be an advantage for tumorigenesis. Second, obesity-related hyperleptinemia and central leptin resistance are promoters of tumor invasiveness and migration. Finally, obesity leads to immune dysfunction, including but not limited to imbalance of macrophages, decreased activity of NK cells, and altered intestinal immunity (Gan et al., 2018). Cachexia is an energy-wasting syndrome that frequently occurs in cancers. In cancer cachexia, many organs, such as bone, brain, liver and gut, as well as tumor tissue, secrete inflammatory factors or pro-cachectic cytokines to promote skeletal muscle wasting (Argiles et al., 2018); whether this process is direct or indirect warrants further investigation. Endocrine and central nervous system perturbations combined with cachexia-related mediators can also elicit catabolic changes in adipose tissues (Baracos et al., 2018).

Recent findings demonstrated system-wide coordination and communication among tissue clocks (Dyar et al., 2018; Koronowski et al., 2019). Peripheral organs harbor their own autonomous circadian rhythms but are synchronized by the hypothalamic suprachiasmatic nucleus via neuro-endocrine pathways (Mohawk et al., 2012). Epidemiological and clinical data confirm a close connection between the disruption of circadian rhythms and hormone-dependent cancers, such as breast cancer and prostate cancer (Masri and Sassone-Corsi, 2018). Cancer can disturb the systemic circadian clock to induce multi-organ chronic inflammation, metabolic disorders, and cachexia by secreting hormones and cytokines (Baracos et al., 2018). A landmark paper showed that lung adenocarcinoma distally rewires circadian transcription and metabolism in the liver but not other organs through altered pro-inflammatory responses via secretion of IL-6, TNF-α, and lactate (Masri et al., 2016) (Figure 2C). However, it remains unclear whether dysregulated liver metabolism further boosts tumor progression in the context. More interestingly, a preclinical study has shed light on the molecular link of circadian disruption with tumorigenesis. Circadian disruption promotes cholestasis, peripheral clock disruption, and sympathetic dysfunction, which lead to activation of the well-known liver tumor promoter, constitutive androstane receptor (CAR), resulting in progression of non-alcoholic fatty liver disease (NAFLD)-induced hepatocarcinogenesis (Kettner et al., 2016). This study connects circadian homeostasis of liver metabolism to tumorigenesis and provides promising candidate strategies for prevention of metabolic syndrome-induced hepatocarcinogenesis by restoration of bile acid homeostasis and inhibition of CAR activation.

### Perineural Invasion and Cancer

Autonomic and sensory nerves innervate and regulate the physiological functions of various types of peripheral organs. The nervous system has also been postulated to play an important role in tumorigenesis. A reciprocal signaling loop between the pancreas and sensory neurons supports inflammation associated with oncogenic Kras-induced neoplasia (Saloman et al., 2016). Aberrant changes in the nervous system, including increase in the size (neural hypertrophy) and number (increased neural density), are profoundly implicated in the process of tumorous deterioration (Demir et al., 2015). Perineural invasion (PNI) is characterized as a course of neoplastic invasion of nerves, which facilitates the crosstalk between cancer cells and nerve fibers and contributes to unconventional tumorigenesis; cancer cells stimulate nerve infiltration in the tumor by the release of neurotrophic growth factors; conversely, nerve endings release neurotransmitters that can activate tumor growth and metastasis through the stimulation of specific membrane receptors (Boilly et al., 2017). PNI is emerging as an important pathologic feature of some malignancies, including the pancreas, colon and rectum, prostate, head and neck, biliary tract, and stomach (Bapat et al., 2011). Autonomic nerve fibers infiltrated in the prostate gland regulates prostate cancer development and dissemination (Magnon et al., 2013). Similarly, vagal innervation promotes gastric cancer cell stemness via M3 receptor-mediated Wnt signaling and denervation suppresses gastric tumorigenesis (Zhao et al., 2014). Recently, an additional mechanism that links PNI to pancreatic ductal adenocarcinoma (PDAC) progression was outlined; PNI is associated with hyperactivation of cholinergic signaling, which reprograms the immune microenvironment through impairing CD8^+^ T cell infiltration and favoring Th2 over Th1 differentiation (Yang et al., 2020). While it is appreciated that the communication between the nervous system and cancers is determinant for tumor development, further studies about the origin of tumor-infiltrating nerves and how they are recruited to tumor tissues and their biological functions is required to determine the nerve dependence of cancers.

### Hormone and Organ-Specific Tumorigenesis

Other examples of organ-specific tumorigenesis are hormones, which are involved in tumor initiation of endocrine organs, including pancreatic cancer, breast cancer, and prostate cancer (Jasienska et al., 2017). Pancreatic cancer is manifested by severe cachexia, marked insulin resistance and diabetes mellitus. Significant loss and dysregulation of Langerhans Islets are present in pancreatic cancer and are likely caused by diabetogenic substances released from tumor cells (Javeed et al., 2015). Therefore, considerable progress suggests that diabetes is secondary to pancreatic cancer. Since diabetes often precedes pancreatic cancer, it is also identified as a risk factor for malignancy. Despite the nature of this issue awaiting further research, there is an opportunity to initiate screening for diabetes to aid early detection of pancreatic cancer (Singhi et al., 2019). In the prostate, androgen receptor (AR) signaling is critically regulated by the hypothalamic–pituitary–testicular axis, adrenal gland steroidogenesis and prostate cell intrinsic factors. Sustained androgen receptor (AR) signaling is the major driver of castration-resistant prostate cancer (CRPC) and promotes prostate cancer development through the regulation of not only transcription networks but also genomic stability and DNA repair (Mills, 2014; Watson et al., 2015). Similarly, most breast cancers are driven by estrogen receptor (ER). Endocrine-related therapies that target ER dependence by selective estrogen receptor modulators have provided substantial improvements in patient outcomes (Turner et al., 2017).

Collectively, these facts inspire us to conclude that tumorigenesis depends on tissue-specific context, which is, not only determined by particular oncogenic mutations, but also tremendously influenced by environmental factors at the organ-level. Moreover, information exchange among organs through neuro-hormone-immune axis dramatically affects tumor progression.

## Regulation at the Tumor Microenvironment Level

### Tumor Microenvironment

**FIGURE 3:**
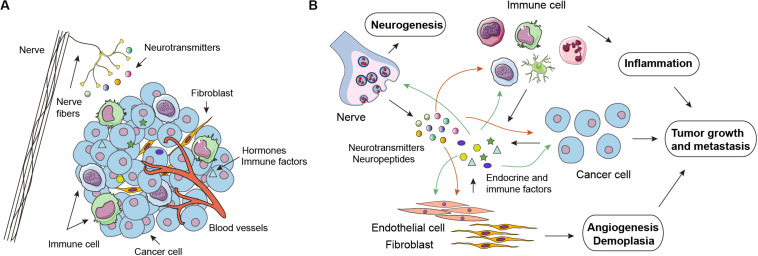
Neuro-endocrine-immune regulations in the tumor microenvironment. **(A)** In addition to cancer cells, the tumor microenvironment (TME) is infiltrated by many other cell types, including cancer-associated fibroblasts (CAFs), endothelial cells (ECs), and various immune cell subsets, such as macrophages, natural-killer cells, dendritic cells, and T cells. Notably, emerging evidence has revealed that infiltrated nerve is an important component of the TME. **(B)** Neurotransmitters (catecholamines and acetylcholine) released by nerve endings and endocrine-immune factors secreted by cancer cells and other cell components in the TME have a stimulatory impact on tumor growth, metastasis, angiogenesis, demoplasia, and inflammation in an autocrine or paracrine manner.

The TME is the primary location for the initiation and progression of cancer, and encompasses multiple cell types and substances (Figure 3A). On the one hand, cancer cells receive signals released by their surrounding stroma, thus promoting cell survival, proliferation, mobility, and other malignant phenotypes. Conversely, cells such as cancer-associated fibroblasts, endothelial cells, and immune cells are functionally sculpted by cancer cells through diverse secreted factors. Reciprocal paracrine interactions between cancer cells and the proximal stromal and immune cells ultimately result in a milieu that favors tumor growth, metastasis, angiogenesis, and evading host immunosurveillance (Figure 3B). Similar to the central- and organ-level regulation, TME is also under the control of the NEI axis. Neurotransmitters, hormones, cytokines, and chemokines are pivotal signaling molecules that implement the microenvironmental regulation of the NEI axis.

### Neural Signaling in Tumor Microenvironment

Similar to the processes of neoangiogenesis and lymphangiogenesis, compelling evidence suggests the possibility of the formation of new nerve fibers within tumor tissues, a phenomenon termed neoneurogenesis, which has been experimentally demonstrated to be important for tumor initiation and progression (Hanoun et al., 2015; Faulkner et al., 2019) (Figure 3A). Neurotransmitters or neuropeptides liberated by nerve fibers in the microenvironment are in the position of stimulating both cancer and stromal cells. Adrenergic neurotransmitters, including norepinephrine (NE) and epinephrine (E), play an important role in the fight-or-flight response by increasing blood flow to muscles, output of the heart, pupil dilation, and blood sugar, and perform their functions by binding to α and β receptors. Recent studies suggest that tumor-specific sympathetic denervation inhibits tumor progression (Magnon et al., 2013), contributes to tumor-induced changes in metabolism (Borniger et al., 2018), and reduces the expression of immune checkpoint molecules, such as programmed death-1 (PD-1), programmed death ligand-1 (PD-L1), and FOXP3 (Kamiya et al., 2019). Previously, we found that monoamine oxidase A (MAOA), the major NE/E degrading enzyme, is significantly downregulated in HCC, and conducts an inhibitory function in NE-induced HCC metastasis via blocking β-AR signaling and EGFR transactivation (Li et al., 2014). Notably, activation of the SNS also stimulates macrophage infiltration and inhibits cellular anti-tumor immune responses indicative of its regulatory role in the immune microenvironment (Cole et al., 2015). Apart from the autonomic nervous system, inhibition of cholinergic signaling and muscarinic receptors is sufficient to suppress gastric tumorigenesis and ablation of sensory neurons has been shown to slow the initiation and progression of pancreatic cancer (Zhao et al., 2014; Saloman et al., 2016). Enhanced cholinergic signaling in pancreatic cancer suppresses cancer cell stemness, CD11b^+^ myeloid cells, TNFα levels, and metastatic growth in the liver (Renz et al., 2018b). Our research has proven that acetylcholine (Ach) can promote the migration and invasion of HCC cells, but inhibit their apoptosis through binding of androgen receptor (Nie et al., 2013). Therefore, it seems plausible that cancer cells can exploit locoregional neural plasticity to facilitate their outgrowth or invasive capacity. Moreover, the vagus nerve is proposed to inhibit colorectal tumorigenesis because of its anti-inflammatory properties; splenic denervation or deletion of the spleen-derived anti-inflammatory peptide TFF2 interrupts the anti-inflammatory neural arc, resulting in the expansion of myeloid derived suppressor cells (MDSCs) and colorectal cancer (Dubeykovskaya et al., 2016). Therefore, it is of great importance to note that there is a tropic relationship between the cancer type and the innervation source. In PDAC, we have also figured out that TME is enriched with tumor-originated 5-hydroxytryptamine (5-HT). More intriguingly, PDAC cells have increased levels of 5-HT receptor HTR2B, which contributes to elevated tumor glycolysis under metabolic stress and promotes the growth of PDAC (Jiang et al., 2017). Pancreatic cancer cells are capable of readily absorbing 5-HT in a transport-mediated manner. The abundant and fast accumulation of intracellular 5-HT greatly activates the small GTPase Ras-related C3 botulinum toxin substrate 1 (Rac1), which is necessary for acinar-to-ductal metaplasia (ADM), a vital process in cancerous transformation (Saponara et al., 2018). 5-HT is also a versatile immunomodulatory molecule in the TME that affects platelet activation and macrophage polarization to accelerate tumor metastasis and dissemination (Jiang et al., 2019). Collectively, the neuro-immune interaction is essential for driving cancer development and progression. The interplay between tumor infiltrating nerves, tumor cells, and non-tumor cells has been largely elucidated. Therefore, the identification of nerve dependence in cancer might prove to be a promising therapeutic opportunity.

### Endocrine-Immune Signaling in Tumor Microenvironment

In addition to neurotransmitters, hormones also affect many vicious behaviors of cancer cells. There are no doubts of the direct promotional effect of estrogens and androgens on tumors of reproduction-related organs (prostate, endometrium, breast and ovary). Estrogens may also induce an immunosuppressive TME through shifting the balance in favor of Th2 responses, proliferation of regulatory T cells and MDSCs, increased PD-L1 expression, and inhibition of the anti-tumor effect of CD8^+^ T cells and NK cells (Svoronos et al., 2017; Welte et al., 2017). Likewise, androgen-mediated suppression of immune reactivity lowers the threshold for cancer and promotes tumor progression (Gubbels Bupp and Jorgensen, 2018). Additionally, multiple known endocrine hormones, such as insulin, prostaglandins, glucocorticoids, prolactin, and progesterone, have been observed to play a role in cancers by modulating malignant phenotypes or anti-tumor immune responses (Klil-Drori et al., 2017; Wang and DuBois, 2018; Dandawate et al., 2019). For instance, inhibition of prolactin receptor signaling by diphenylbutylpiperidine antipsychotic drugs reduces the growth of PDAC (Dandawate et al., 2019); prostaglandin regulates tumor-associated immunosuppression by inducing MDSC and Treg differentiation, macrophage polarization from M1 to M2, and production of PD-L1 (Wang and DuBois, 2018). Our group found that mineralocorticoid receptor expressed in hepatocellular carcinoma suppresses cancer progression by regulating the miR-338-3p-PKLR axis and the Warburg effect (Nie et al., 2015). Notably, an obvious example of an indirect effect is that upregulated thyroid hormones, stimulated by acute or chronic stress, are involved in tumor evolution by alternating T-cell lymphoproliferative responses, implicating the role of the neuron-endocrine-immune cascade in modulating tumor progression. Therefore, targeted inhibition of hormone-related signaling may act as a novel strategy to eliminate tumor cells within the TME and enhance the effects of immunotherapies.

## Conclusion

In this review, we have focused on the interrelationships between NEI factors and cancer initiation and metastasis and proposed that cancer is a systetic disease influenced by multidimensional regulation: central-level regulation, organ-level regulation, and TME-level regulation. At each level, nervous system, endocrine, and immune factors play a critical role. At the central-level of regulation, emotions play essential roles in the growth, angiogenesis, and metastasis of cancer, as chronic stress promotes cancer progression, while the enriched environment (positive attitude, exercise) may hamper this process. Organ-level modulations range from changes in whole-body homeostasis to alterations in pathological conditions such as cancers. The TME contains various cellular types executing concrete functions, under the regulation of signaling molecules including neurotransmitters, hormones, cytokines/chemokines, and other types of secreted proteins. Although the roles of the nervous, endocrine, and immunes system in tumor initiation and progression have been investigated in recent decades, the knowledge is too detailed and scattered to easily understand the systemic contribution. Hence, our proposal of systemic regulation may provide novel avenues for a better understanding of mechanisms in carcinogenesis and development, digging out sensitive biomarkers for diagnosis and effective therapeutic interventions. Moreover, it provides a certain theoretical basis for studying the impact of social and mental factors on cancer patients, and improving the comprehensive treatment of postoperative rehabilitation of cancer patients including social family care and psychological counseling.

From the preventative and therapeutic points of view, it is also worth noting that a variety of inhibitors targeting the NEI axis have been widely used in clinical practice, such as beta-blockers, Ach receptor antagonists, and 5-HT receptor inhibitors. For example, beta-blockers are currently prescribed for cardiovascular diseases, but many retrospective studies have revealed a beneficial effect in patients with prostate cancer (Grytli et al., 2014), colorectal cancer (Cui et al., 2019), and multiple myeloma (Hwa et al., 2017). It is unclear how beta blockers improve patient survival and impede tumor progression. Further research is warranted to fully uncover the complexity of the mechanisms along with the interplay with endocrine and immune systems. Therefore, our hypothesis further support the repurposing of already approved drugs, which not only minimizes time and costs, but also may prove to be an innovative therapies in oncology.

Finally, we emphasize that the role of systemic regulation of tumors does not negate the importance of local lesions, as local lesions and the whole body are interdependent. For early and mid-term malignant tumors, whether treatment involves surgery, chemotherapy, or local interventional therapy, the removal of tumor lesions is conducive to the functional repair of the overall regulatory system. Importantly, cancer treatment should be based on the premise of not sacrificing the activity of the body’s regulatory system, and more attention should be paid to the detection of systemic markers, including markers of nervous, endocrine, and immune systems.

## Author Contributions

All authors listed have made a substantial, direct and intellectual contribution to the work, and approved it for publication.

## Conflict of Interest

The authors declare that the research was conducted in the absence of any commercial or financial relationships that could be construed as a potential conflict of interest.
